# Chronic Effects of Foam Rolling on Flexibility and Performance: A Systematic Review of Randomized Controlled Trials

**DOI:** 10.3390/ijerph19074315

**Published:** 2022-04-04

**Authors:** Jeffrey Cayaban Pagaduan, Sheng-Yuan Chang, Nai-Jen Chang

**Affiliations:** 1 Institute of Active Lifestyle, Palacký University, Olomouc 77200, Czech Republic; jcpagaduan@gmail.com; 2 Department of Industrial Education, National Taiwan Normal University, Taipei 106, Taiwan; zl1451@ms1.nihs.tp.edu.tw; 3 Office of Physical Education, Taipei Municipal Nei-Hu Vocational High School, Taipei 114, Taiwan; 4 Department of Sports Medicine, Kaohsiung Medical University, Kaohsiung 807, Taiwan; 5 Department of Medical Research, Kaohsiung Medical University Hospital, Kaohsiung 807, Taiwan; 6 Ph.D. Program in Biomedical Engineering, College of Medicine, Kaohsiung Medical University, Kaohsiung 807, Taiwan

**Keywords:** self myofascial release, foam rolling, range of motion, athletic performance, exercise

## Abstract

The purpose of this study is to review the existing literature on chronic effects of foam rolling (FR) on flexibility and performance. Electronic databases were searched during January 2022 for topics related to FR. Included studies met the following criteria: (a) peer-reviewed articles written in English; (b) FR intervention of at least four weeks; (c) non-motorized FR device during intervention; (d) randomized controlled trial with existence of a control group; and (e) any lower body parameter related to flexibility, recovery, and performance. Nine studies met that criteria. Results revealed that chronic FR demonstrated conflicting results for improvement of flexibility. On the other hand, a majority of the articles in this review showed no beneficial effects of FR on performance. Lastly, the effect of FR on recovery is unclear. These findings suggest the need for further studies to establish the consensus about the long-term application of FR in flexibility, recovery, and performance.

## 1. Introduction

Foam rolling (FR) is a self-massage technique utilizing a tool and applying pressure to the muscle and fascia. The FR is typically performed before and after an exercise activity. In rehabilitation settings, FR is implemented within the strength training regime. Different FR tools are used, ranging from types of roller massage with varying densities, and to some extent, inclusion of motorized technology that creates vibration with FR [1,2]. Researchers recommend one to three sets of FR, with rolling durations of 30–120 s per set to attain the benefits of FR [3]. There has been an increasing interest towards FR among individuals from clinical and non-clinical populations.

Researchers propose various mechanisms for improving ROM and performance with FR. The first is the modulation of pain in the central nervous system using FR. The constant pressure exerted on the soft tissues overload the skin receptors, inhibiting pain sensation and stretch tolerance [4,5]. This mechanism is supported by previous studies demonstrating improvement in pain sensation with FR [6,7]. Another mechanism is related to increased blood flow and reducing the incidence of fascial inflammation or tightness of fascia as a result of inflammation [8]. These two mechanisms may also contribute to performance enhancement. For example, the reduced pain perception and increased blood flow with FR may influence the recovery of muscle function, which may play an important role in performance. In addition, the application of FR may accelerate the healing of the muscle by increasing the circulating neutrophils [9]. The FR may also facilitate an increase in alphamotor neuron activity and output and lower neural inhibition, thereby allowing better communication of the afferent receptors in the connective tissue [10,11]. Other mechanisms with FR include improvement in skeletal muscle oxygenation [12] and parasympathetic activation [13]. Thus, the potential benefits of FR on flexibility and performance are linked to different physiological mechanisms. 

Systematic reviews were carried out to examine the short-term effects of FR on flexibility, recovery, and performance related to muscular contractions. A majority of the reviews demonstrated an increased range of motion (ROM) from acute FR [2,4,14,15,16,17,18]. Some also reported alleviation of muscle soreness [14,16], pain sensation [2,16], and muscle stiffness [16] with FR. However, FR posted contrasting outcomes with regard to recovery [2,15,18]. Non-enhancement of performance with FR were also documented [1,2,15,18] [ While these reviews highlight the gaining popularity of FR in athletic, recreational, and rehabilitation settings, a majority of the reviews examined acute settings.

Currently, there has been a scarcity in the literature investigating the long-term effects (≥4 weeks) of FR on flexibility, recovery, and performance. Further, the combination of different FR devices from the existing literature may fail to delineate device-specific FR adaptations. These gaps create a void in translation from research to practical application, utilising FR to individuals within performance enhancement settings. Thus, the purpose of this systematic review was to examine the long-term effects of FR on flexibility and performance.

## 2. Materials and Methods

### 2.1. Eligibility Criteria

The selection of articles for inclusion in this systematic review were based on the following criteria: (1) publications appearing in peer-reviewed journals, written in English; (2) FR intervention administered for at least four weeks; (3) studies conducted using randomized controlled trial, with the presence of a control group (CON); (4) only non-motorized foam roller used as a device for FR intervention; and (5) availability of any lower body measure for flexibility, recovery, and performance.

### 2.2. Information Sources and Search Strategy

A systematic search strategy by S.Y.C. was conducted in accordance with the Preferred Reporting Items for Systematic Reviews and Meta-analyses (PRISMA) guidelines for reporting systematic reviews [19]. The literature was considered if it was published up until January 2022 and administered using PubMed, ProQuest, SPORTDiscus, ScienceDirect, and SpringerLink. Search terms were combined using the Boolean phrase “foam rolling” or “self myofascial release” and “foam roller massage” or “myofascial pain” or “pressure pain threshold” or “range of motion” or “delayed onset of muscle soreness” or “muscle damage”. The reference lists of selected studies were also searched for potential inclusion of other studies. The protocol for this review was registered to the International Prospective Register of Systematic Reviews (PROSPERO; Registration No. CRD42022306039).

### 2.3. Quality Check

The included studies in this systematic review were assessed using the Physiotherapy Evidence Database Scale for appraising the quality of the literature. Any disagreement between J.C.P. and N.J.C were discussed; however, if unresolved, it was settled by a third reviewer.

Each study underwent methodological quality assessment using the Physiotherapy evidence database (PeDro) scale [16]. The PeDro is comprised of 11 criteria, with a score of 1 awarded for meeting a criterion. A study is considered either high quality (6–11 points), low quality (4–5), or poor (0–3).

### 2.4. Characteristics of Studies

The studies included in the review were extracted into Excel with the following details: author, participants, intervention duration, protocol, measures, and results.

## 3. Results

**Figure 1:**
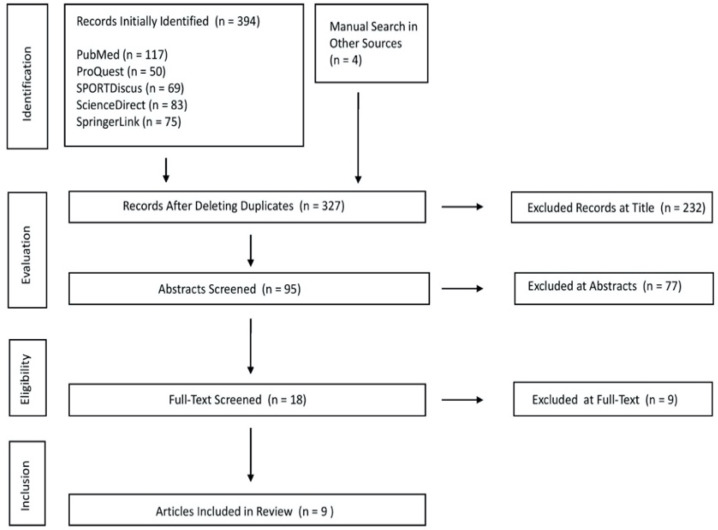
PRISMA search strategy.

A total of 328 articles were initially identified from the literature search. Of those, 319 were excluded for not meeting the criteria, with the reasons for exclusion outlined in Figure 1. Nine studies were eventually included in the review [20,21,22,23,24,25,26,27,28].

### 3.1. Quality Appraisal

All nine studies demonstrated a high quality of evidence. No study was able to satisfy the concealment and blinding criteria for participants (Question 5) or blinding of intervention facilitators (Question 6) and assessors (Question 7). The nature of the study may have contributed to the difficulty in meeting the criteria related to blinding. The mean PeDro score for the studies included in the review was 6.33 ± 0.50. There were six studies that scored 6 and three studies that scored 7. Table 1 displays the PeDro scores of the methodological quality of included studies.

### 3.2. Sample Population

Two studies [20,21] included highly-trained athletes. One study involved male and female soccer athletes [20], whereas the other study [21] recruited male rugby players. Two studies employed recreationally-active males [24,26] and three studies [22,23,26] involved recreationally-active males and females. Two studies were conducted in healthy males and females [25,26,27]. One study included patients with hip osteoarthritis [28]. Eight studies [20,21,22,23,24,25,26,27] employed young adults and one study was carried out in middle-aged adults [28].

### 3.3. Intervention

A variety of FR interventions were found in all the studies that included a physical activity routine, except for one study that implemented a home exercise program [28]. Three studies employed FR on a single muscle [20,24,25,26,27], the gastrocnemius [20,25,27], and two studies employed FR for hamstring muscles [24,26]. Two studies utilized FR on two muscles, one for quadriceps and hamstrings, and one for the gluteus and quadriceps. Two studies applied FR on multiple muscle groups [21,23]. In terms of FR intervention duration, four studies lasted for four weeks [20,22,23,26], one was facilitated for five weeks [25], one study was carried out for 6 weeks [21], one study was employed for 8 weeks [8], and one study lasted for 11–12 weeks [28].

### 3.4. Control

Different CON groups were identified in the included studies. Three studies involved a specific exercise routine [20,21,28], and two studies utilized a specific stretching activity [26,27], while maintaining a physical activity routine. Four studies maintained a regular exercise/training routine [22,23,24,25].

### 3.5. Effects of FR on Flexibility

Eight out of nine studies utilized at least one flexibility index to determine the chronic effect of FR on flexibility [20,21,22,23,24,25,26,27]. Three studies employed the ankle dorsiflexion ROM for measurement of flexibility [20,25,27]. One study used passive knee extension [26]. Guillot et al. [21] administered side splits, active/flexed leg raises, and hip extensions to measure flexibility. Two studies assessed flexibility from the stand-and-reach test [23,24]. Guillot et al. [21] used FR for six weeks and Junker & Stöggl [24] for eight weeks. Results showed conflicting outcomes, with five out of eight studies displaying beneficial outcomes on flexibility from chronic FR [20,21,23,24,25]. Aune et al. [20] exhibited increased ROM after a daily gastrocnemius FR for three sets of 60 s. Similarly, Guillot et al. [21] posted increased ROM from a single set of 20/40 s FR for hip extensors, hip flexors, knee extensors, and knee flexors. Junker & Stöggl [23] recorded ROM improvement after a thrice a week, three sets of 30–40 s hamstring FR. Junker & Stöggl [24] showed an enhancement in ROM from twice a week, unilateral/bilateral FR (three × 30–50 s) for quadriceps, hamstrings, glutes, illiotibial band, and calf muscles. Lastly, Kiyono et al. [25] presented increased ROM after a thrice a week unilateral FR (three sets × 30 s).

### 3.6. Effects of FR on Performance

Six out of nine studies measured at least one performance outcome from FR [20,21,24,25,26,28]. Aune et al. [20] employed plantar flexor torque and drop jump for measuring performance. Hodgson et al. [21] tested neuromuscular efficiency during a lunge, single leg countermovement jump, knee flexor and extensor maximal voluntary isometric contraction, and pain pressure threshold of biceps femoris and rectus femoris. Ikutomo et al. [28] assessed performance using a hip pain visual analog scale, Haris Hip Score, and the Japanese Orthopedic Association Hip-Disease Evaluation Questionnaire (JHEQ). Junker & Stöggl [24] used the Bourban Trunk Muscle Strength Test, standing long jump, single-leg triple hop, and Y-Balance Test. Kiyono et al. [25] measured the dorsiflexion ROM passive torque and muscle stiffness. Morton et al. [26] utilized the peak passive knee torque and muscle stiffness. Out of the six studies, only the study of Ikutomo et al. [28] demonstrated improvement in performance from reduction in hip pain, and higher Haris Hip and JHEQ scores. The FR was administered for 11–12 weeks in patients with osteoarthritis, and FR intervention included a home exercise 10-min FR program for the gluteus and the hamstring and quadricep of the symptomatic leg [28].

### 3.7. Effects of FR on Recovery

None of the included studies for systematic review exhibited any marker related to recovery. Table 2 displays the characteristics of studies included in the review.

## 4. Discussion

This systematic review assessed the chronic effects of FR on flexibility, performance, and recovery from nine studies, implemented for at least four weeks. Eight studies were included for investigating the effect of FR on flexibility, while six studies were examined to determine the effect of FR on performance. Results revealed non-conforming effects of FR on flexibility, with a majority of the studies exhibiting increased flexibility with FR. On the other hand, most of the included studies in the review demonstrated non-significant differences in performance between FR and CON. Lastly, there was no study that investigated recovery.

### 4.1. Effects of Foam Rolling on Flexibility

With respect to the long-term benefits of FR on flexibility, a majority of included studies have reported that FR can increase joint range of motion (ROM) [20,21,24,25]. In particular, the intervention frequency necessary to improve ROM is usually three times per week, with each intervention session comprising three, 30–50 s sets. It is believed that FR influences flexibility by removing the limitation of soft tissue adhesion and consequently increasing the extensibility of target muscles [15]. During FR, bodyweight is used to apply pressure, resulting in the transmission of sensations through the peripheral pressure receiver to the central nervous system, thus regulating pain tolerance. However, one study [22] reported that a FR intervention did not improve hip flexion ROM, despite the similarity in protocol with the aforementioned studies [21,23,24,25]. One possible reason for this non-conforming result is the utility of the roller massager to the thigh muscles [22], with pressure from upper limb actions. This may not be sufficient to achieve the desirable force to produce improvement in flexibility. In addition, three studies [20,25,27] have investigated the influence of FR on ankle dorsiflexion ROM, with only one reporting improved dorsiflexion ROM [25]. The difference in results could be related to the variety of testing methods implemented. Kiyono et al. [25] used an isokinetic equipment, with the knee measured at a 0° angle. One theory for the enhancement of flexibility is linked to stretch tolerance. The force exerted by FR on the muscles to trigger the transmission of messages to reduce pain sensation and increase stretch tolerance may not be sufficient to facilitate improvement in flexibility [2,20]. On the other hand, the other two studies [20,27] that did not report any significant difference in dorsiflexion ROM enhancement with the control performed measurements through lunge tests. This may partially explain the non-difference as the lunge movement mainly targets the soleus muscle, but FR primarily focused on the gastrocnemius [20,27]. The non-coherent findings of long-term FR in this review are due to variability in FR protocols and testing methods for assessment of flexibility.

At present, the benefits of FR on flexibility are primarily related to the acute neural response, with optimal results achieved within 2 min after intervention, and the beneficial effects subsiding after approximately 30 to 60 min [14]. The rapid decline of neural response is caused by rolling friction-induced increases in temperature of the skin [6], the reduction of the H reflex [29], and corticospinal excitability [6,30] that occurs during FR. Both measures return to their reference points at the conclusion of an FR session [22]. Furthermore, although there is no robust evidence that FR releases myofascial [31], immediate increases in soft tissue structure (e.g., soft tissue elasticity, muscle stiffness), tolerance to stretch (e.g, pain pressure threshold) has also been observed [15,25,32]. On the other hand, long-term increases in ROM are assumed by changes in pain perception rather than actual changes in soft tissue structure [3,14,25].

The current methods for evaluating flexibility are mostly focused on joint ROM and limb reach distance (length), and these methods cannot independently evaluate factors relating to soft tissue adhesion. This hypothesis can be verified by incorporating additional examinations into the testing process, using muscle tension testing equipment, soft tissue ultrasounds, and bioimpedance analyses. In addition, temperature may also be a factor that affects blood flow [4]. These parameters warrant further investigation to elucidate crucial information on the influence of FR on flexibility.

### 4.2. Effects of Foam Rolling on Performance

In this review, a majority of the studies demonstrated no difference in performance with FR compared to CON. Only one study reported long-term improvements in physical performance with the use of FR [28]. In that study, FR was applied to patients with degenerative hip osteoarthritis, with FR applied on the hip and thigh muscles for 10 min, every day for 11–12 weeks. Patients reported significantly reduced hip pain and improved physical function. The pressure created through their own bodyweight with FR may have facilitated soft tissue rearrangement in the hip region, increased the blood flow and circulation, and regulated the perception of pain in the central nervous system [2]. It may also be possible that FR facilitated better communication of the afferent receptors in the connective tissue [10,11]. In addition, FR may have contributed to achieving psychological relaxation [13]. The aforementioned benefits of FR reduced the external discomfort experienced by patients with degenerative osteoarthritis. Although the findings regarding the duration of FR use are inconclusive, the recommended FR duration for reducing pain is approximately 90 s [2]. While long-term benefits of FR exhibited non-significant performance enhancement in healthy populations, FR seemed to demonstrate no adverse effects. Caution must be taken into consideration when using long-duration FR (>9 min) for performance enhancement as this may inhibit nerve excitability and decrease muscle force output [33]. Including a survey for subjective indices in future studies can help identify psychological effects of FR. Furthermore, adding participant adherence monitoring schemes may help link alteration in performance with FR. FR can immediately improve joint stretch tolerance and pain control and also increase joint ROM during exercise activities [4]. However, these benefits may have a negligible effect on sports performance or be undetectable in selective measurements [2]. Thus, more studies are needed to establish the role of chronic FR in performance settings.

#### Effects of FR on Recovery

No available study investigated any recovery marker from long-term FR. Such findings warrant an inclusion of additional recovery markers in chronic FR studies.

### 4.3. Limitations

The results of the systematic review provide evidence of the chronic effects of FR on flexibility and performance. However, several limitations are noted in this review. First, a majority of the studies include a small sample size, which may result in low power. Second, most of the studies fail to administer blinding, thereby increasing the bias for a placebo effect.

Third, the studies involved different populations, contributing to large variability in results. These limitations may explain the incoherent findings in the review and should be accounted for in future studies. 

## 5. Conclusions

This systematic review demonstrated no detrimental effect of long-term FR on improving flexibility. However, a consensus on the chronic effect of FR of flexibility was not achieved. The findings on chronic FR warrant the need for further investigation to identify potential mechanisms that may help explain the dose–relationship of FR and flexibility. Additionally, while a majority of the studies in this review demonstrated non-effects of FR on performance, long-term FR does not appear to be harmful in performance enhancement settings. Lastly, there is no information with regard to the effect of chronic FR on recovery.

## Figures and Tables

**Table 1:** PeDro Score of Studies Included in the Systematic Review.

Study	Item											
	1	2	3	4	5	6	7	8	9	10	11	Total
Aune et al. 2018 [20]	1	1	0	0	0	0	0	1	1	1	1	6
Guillot et al. 2019 [21]	1	1	0	0	0	0	0	1	1	1	1	6
Hodgson et al. 2018 [22]	1	1	0	0	0	0	0	1	1	1	1	6
Ikutomo et al. 2020 [28]	1	1	0	0	0	0	0	1	1	1	1	6
Junker & Stöggl, 2015 [24]	1	1	1	0	0	0	0	1	1	1	1	7
Junker & Stöggl, 2019 [23]	1	1	0	0	0	0	0	1	1	1	1	6
Kiyono et al. 2020 [25]	1	1	0	1	0	0	0	1	1	1	1	7
Morton et al. 2016 [26]	1	1	0	0	0	0	0	1	1	1	1	6
Smith et al. 2019 [27]	1	1	1	0	0	0	0	1	1	1	1	7

Item 1: Eligibility criteria; Item 2: Random allocation of subjects; Item 3: Allocation concealment; Item 4: Similarity of intervention groups; Item 5: Blinding of subjects; Item 6: Blinding of intervention trainers; Item 7: Blinding of assessors; Item 8: 85% of the subjects initially allocated completed at least one key outcome; Item 9: All subjects received treatment or control with an “intention to treat”analysis performed; Item 10: Between group comparison executed for at least one variable; and Item 11: Study provides both point measures and measures of variability for at least one key outcome.

**Table 2:** Chronic Effects of FR on Flexibility and Performance.

Author	Participants		Duration	Protocol		Measures		Result	
	FR	CON	Flexibility	Performance	Flexibility	Performance			
Aune et al. 2018 [20]	23 male and female top-division soccer players		4 weeks	daily FR on gastrocnemius (3 × 60 s)	daily single-leg heel drop exercise (3 × 15 repetitions)	DFROM	maximal isometric plantar flexion torque drop RSI	DFROM: FR ↔ CON	plantar flexion torque: FR ↔ CON drop RSI: FR ↔ CON
Guillot et al. 2019 [21]	30 male professional rugby players		6 weeks	FR20: unilateral FR for hip extensors, hip adductors, knee extensors, and plantar flexors (1 × 20 s for 15 sessions) FR40: same FR intervention with FR20 but executed for 1 × 40 s	cycling task at 50% Vo2Max	side split active flexed/straight leg raise hip extension	not available	All dependent variables: FR20 > CON FR40 > CON	not available
CON–control group; Vo2Max–maximal oxygen consumption; DFROM–dorsiflexion range of motion; RSI–reactive strength index.									
**Author**	**Participants**	**Duration**	**Protocol**		**Measures**			**Result**	
			FR	CON	Flexibility	Performance		Flexibility	Performance
Hodgson et al. 2018 [22]	23 recreationally -active males and females	4 weeks	FR3: 3 days/week of alternating FR for quadriceps and hamstrings for 4 × 30 s FR6: same FR protocol with FR3 but performed for 6 days/week	regular training routine	active and passive hip flexion ROM	neuromuscular efficiency during a lunge single leg CMJ knee flexor and extensor MVIC pain pressure threshold of biceps femoris and rectus femoris		active and passive hip flexion ROM: FR3 ↔ CON FR6 ↔ CON	all dependent variables: FR3 ↔ CON FR6 ↔ CON
Ikutomo et al. 2020 [28]	74 male and female patients with osteoarthritis	11–12 weeks	home exercise program with 10-min FR for gluteus, hamstrings, and quadriceps of the affected leg	home exercise program	not available	hip pain VAS Haris Hip Score JHEQ		not available	hip pain VAS: FR < CON Haris Hip Score and JHEQ: FR > CON
Junker & Stöggl 2015 [24]	26 recreationally- active males	4 weeks	3 days/week of 3 × 30–40 s hamstring FR for both limbs	regular training routine	Stand-and- Reach Test	not available		Stand-and-Reach Test	Stand-and -Reach Test: FR > CON
MVIC–maximal voluntary isometric contraction; VAS–visual analog scale; JHEQ–Japanese Orthopedic Association Hip Disease Evaluation Questionnaire.									
**Author**	**Participants**	**Duration**	**Protocol**		**Measures**			**Result**	
			FR	CON	Flexibility	Performance		Flexibility	Performance
Junker & Stöggl 2015 [23]	25 recreationally- active males and females	8 weeks	Twice a week, unilateral/bilateral FR for gluteus, quadriceps, hamstrings, illiotibial band, and calf muscles (3 × 30–50 s)	regular training routine	Stand-and-Reach Test	Bourban Trunk Muscle Test standing long jump single leg, triple hop for distance Y-Balance Test knee flexor and extensor MVIC		Stand-and-Reach Test: FR > CON	all dependent variables: FR ↔ CON
Kiyono et al. 2020 [25]	30 healthy males and females	5 weeks	thrice a week, unilateral FR for gastrocnemius (3 × 30 s)	regular training routine	DFROM	DFROM passive torque Haris Hip Score JHEQ muscle stiffness		not available	Haris Hip Score and JHEQ: FR > CON DFROM passive torque and muscle stiffness: FR6 ↔ CON
**Author**	**Participants**	**Duration**	**Protocol**		**Measures**			**Result**	
			FR	CON	Flexibility	Performance		Flexibility	Performance
Morton et al. 2016 [26]	20 recreationally- active males	4 weeks	once a day, bilateral FR for hamstrings (4 × 60 s) followed by static stretching of hamstrings (4 × 45 s),	once a day static stretching of hamstrings (4 × 45 s)	passive knee extension ROM	knee extensor MVIC		Passive knee extension ROM: FR ↔ CON	knee extensor MVIC: FR ↔ CON
Smith et al. 2019 [27]	44 male and female university students	6 weeks	FR: twice a week, unilateral FR for gastrocnemius (3 × 30 s) FR + SS: FR with twice a week, static stretching for gastrocnemius (3 × 30 s)	Twice a week, static stretching for gastrocnemius (3 × 30 s)	DFROM	not available		DFROM: FR ↔ CON FR + SS ↔ CON	not available

## Data Availability

Not applicable.
